# Adoption of Large Language Model AI Tools in Everyday Tasks: Multisite Cross-Sectional Qualitative Study of Chinese Hospital Administrators

**DOI:** 10.2196/70789

**Published:** 2025-04-01

**Authors:** Jun Chen, Yu Liu, Peng Liu, Yiming Zhao, Yan Zuo, Hui Duan

**Affiliations:** 1 Medical Services Management Department Peking University People's Hospital Beijing China; 2 Department of Obstetrics and Gynecology Peking University Shenzhen Hospital Shenzhen China; 3 Department of Gynecology and Obstetrics Nursing West China Second University Hospital, Sichuan University Chengdu China; 4 West China School of Nursing Sichuan University Chengdu China; 5 Key laboratory of Birth Defects and Related Disease of Women and Children (Sichuan University) Ministry of Education Chengdu China; 6 School of Public Administration and Policy Renmin University of China Beijing China

**Keywords:** large language model, artificial intelligence, health care administration, technology adoption, hospital administrator, qualitative study, barriers to adoption

## Abstract

**Background:**

Large language model (LLM) artificial intelligence (AI) tools have the potential to streamline health care administration by enhancing efficiency in document drafting, resource allocation, and communication tasks. Despite this potential, the adoption of such tools among hospital administrators remains understudied, particularly at the individual level.

**Objective:**

This study aims to explore factors influencing the adoption and use of LLM AI tools among hospital administrators in China, focusing on enablers, barriers, and practical applications in daily administrative tasks.

**Methods:**

A multicenter, cross-sectional, descriptive qualitative design was used. Data were collected through semistructured face-to-face interviews with 31 hospital administrators across 3 tertiary hospitals in Beijing, Shenzhen, and Chengdu from June 2024 to August 2024. The Colaizzi method was used for thematic analysis to identify patterns in participants’ experiences and perspectives.

**Results:**

Adoption of LLM AI tools was generally low, with significant site-specific variations. Participants with higher technological familiarity and positive early experiences reported more frequent use, while barriers such as mistrust in tool accuracy, limited prompting skills, and insufficient training hindered broader adoption. Tools were primarily used for document drafting, with limited exploration of advanced functionalities. Participants strongly emphasized the need for structured training programs and institutional support to enhance usability and confidence.

**Conclusions:**

Familiarity with technology, positive early experiences, and openness to innovation may facilitate adoption, while barriers such as limited knowledge, mistrust in tool accuracy, and insufficient prompting skills can hinder broader use. LLM AI tools are now primarily used for basic tasks such as document drafting, with limited application to more advanced functionalities due to a lack of training and confidence. Structured tutorials and institutional support are needed to enhance usability and integration. Targeted training programs, combined with organizational strategies to build trust and improve accessibility, could enhance adoption rates and broaden tool use. Future quantitative investigations should validate the adoption rate and influencing factors.

## Introduction

Large language model (LLM) artificial intelligence (AI) tools have gained widespread attention across various industries [1-3]. Tools such as ChatGPT and their counterparts developed in China have demonstrated the ability to interpret textual contexts and respond effectively to user prompts [4]. These tools are increasingly used for tasks such as content generation and answering queries and demonstrate potential in professional environments [5-7].

In health care administration, hospital administrators manage tasks such as document creation, resource allocation, and communication as daily routines, all of which require efficiency and precision. LLM AI tools present opportunities to streamline tasks by providing instant feedback, easy access to information, and improved language suggestions [8,9]. These tools have garnered interest among administrators seeking to enhance personal productivity [5]. However, the complexity of administrative roles, especially in large institutions, poses unique challenges. Administrators often navigate intricate schedules, analyze data, and manage operational demands, whereas LLM AI tools can aid in finding relevant information and generating structured reports [10,11]. Despite such potential benefits, users in China have shown varied interest in adopting these technologies [12,13].

LLM AI tools are transforming health care by enhancing clinical decision-making, streamlining workflows, and improving patient outcomes [14,15]. However, most existing studies focus on clinical applications or formal integration into organizational workflows, leaving a gap in understanding individual-level adoption among administrators [8,16-18]. Personal factors, such as technological confidence, play a critical role in shaping the willingness to use digital tools [19-21]. While early adopters have stressed both enablers and barriers to using LLM AI tools, there is limited evidence exploring such experiences within the administrative context [22-24].

Descriptive qualitative research offers a valuable approach to uncovering the reasons behind the adoption or avoidance of LLM AI tools in administrators’ daily routines [25,26]. Unlike formal organizational protocols, individual adoption often occurs at a personal pace, influenced by preferences, concerns, and specific task requirements. This study focuses on understanding everyday use rather than official implementation, providing insights into the practical enablers and obstacles administrators face.

By examining user experiences among hospital administrators in multiple hospitals in China, this study addresses a critical knowledge gap. The findings can illustrate how LLM AI tools are integrated into day-to-day routines or why they are rejected, offering actionable insights for technology developers and policy makers. The results can inform strategies to better align AI tools with user needs and preferences and lay the groundwork for future research and broader adoption efforts.

## Methods

### Study Settings

The study was conducted across 3 tertiary hospitals in different regions of China, selected for their geographic diversity and varied institutional characteristics.

One leading tertiary hospital in Beijing (site 1), the capital city, the country’s political center, and one of the cities with an abundance of health care resources, 1 tertiary hospital in Shenzhen (site 2) on the eastern coast of China, a city renowned for technological innovation and rapid modernization but known to be in a relative shortage of health care resources, and another tertiary hospital in Chengdu (site 3), Southwest China, which is the regional center in terms of economic development and health care.

Sites 1 and 2 are comprehensive care providers and site 3 is specialized in woman and child health care. All 3 hospitals serve a broad mix of urban and rural populations and reflect a balance between traditional practices and modern approaches to health care management. This diversity in settings allowed the study to capture a wide range of perspectives on the adoption of LLM AI tools in administrative contexts.

### Participant Roles

Participants were hospital administrators engaged in operational and administrative roles, including department managers and coordinators. We excluded clinical staff and senior hospital leaders to focus on middle-level administrators directly involved in routine decision-making and task management. We excluded frontline clinical staff to concentrate solely on administrative perspectives and omitted senior hospital leaders because their strategic roles and varied workflows differ significantly from those of middle-level administrators.

### Study Design

We used a multicenter, cross-sectional, descriptive qualitative design to explore the individual-level adoption of LLM AI tools among hospital administrators. This design was chosen for its ability to capture detailed, context-specific insights into participants’ lived experiences and perceptions, which allowed for flexibility in exploring factors influencing the adoption or rejection of LLM AI tools. The multicenter and cross-sectional elements ensured diverse perspectives across different geographic and institutional contexts [27].

### Time Frame

Data collection was conducted from June 11 to August 16, 2024, across all sites.

### Study Organization

The study was organized collaboratively across the 3 sites. Each hospital had a local principal investigator responsible for participant recruitment and data collection. The local principal investigators were trained through web-based workshops to ensure methodological standardization and consistency. A unified interview guide, developed and piloted collectively by the principal investigators, was used across all sites. Regular web-based meetings were held every 2 weeks to review progress, discuss data analysis, address methodological challenges, and promote reflexivity to ensure the rigor of the study.

### Sampling Strategy

Purposive sampling was used to recruit participants with relevant experiences. This method allowed the inclusion of participants with varied adoption behaviors, including those who stopped using the tools after initial trials. The following criteria were used to select participants.

#### Inclusion Criteria

The inclusion criteria were as follows:

- Aged 26-60 years.
- Both sexes were acceptable.
- Active involvement in administrative decision-making or operations and at least 1 year of work experience in administrative roles.
- Exposure to LLM AI tools: Having used at least 1 LLM AI tool at least once (even if they discontinued use).
- Willingness to provide informed consent.

#### Exclusion Criteria

The exclusion criteria were as follows:

- Individuals without any exposure to LLM AI tools.
- Senior leaders or frontline clinical staff, as their roles differ significantly from the study's focus.
- Inability to provide consent.

Efforts were made to ensure the diversity of participants. In addition to roles such as department managers, coordinators, and frontline administrative staff to ensure that diverse administrative functions were reflected, diversities in terms of age, sex, work experience, education level, and experience in using LLM AI tools were considered when enrolling participants.

### Sample Size

The sample size was determined using the data saturation principle [28]. For this multisite study, data saturation was considered achieved on a per-site basis, when no new themes or insights emerged from subsequent interview transcripts during analysis. The achievement of data saturation was collectively assessed during the regular principal investigator meetings using an iterative analysis process. Local transcripts were continuously reviewed during the meetings. Once the emergent themes or insights became repetitive and no new information was obtained at a given site, we considered that site to have achieved data saturation. This site-specific approach was adopted to accommodate local contextual differences observed throughout the interview process.

### Recruitment Process

Local principal investigators selected and invited participants through direct outreach using their knowledge of the administrative structures and personnel at their respective sites.

### Data Collection

Face-to-face semistructured interviews were conducted to collect data. This technique allowed researchers to probe participants’ responses and capture insights by fostering rapport and encouraging participants to share their experiences openly [28].

The interview guide was collaboratively developed and piloted by the local principal investigators to ensure consistency and relevance. Key domains included participants’ experiences with LLM AI tools, perceived benefits and challenges, and reasons for adoption or rejection. Notably, the interviews incorporated questions designed to assess participants’ levels of familiarity with LLM AI tools and their frequency of use, as defined in Tables 1 and 2, respectively.

Each interview lasted approximately 30–45 minutes and was conducted in Mandarin Chinese. With participants’ consent, interviews were audio-recorded and transcribed verbatim within 48 hours by the local principal investigators to ensure accuracy and timeliness for data analysis. Field notes were taken during or immediately after the interviews to capture contextual details, nonverbal cues, and any additional observations that could enrich the data (Multimedia Appendix 1).

**Table 1:** Levels of participant’s familiarity of LLM^a^ AI^b^ tools.

Level of familiarity	Definition
High	Participant demonstrates a strong understanding of LLM AI tools, including advanced features and applications; has used the tools for multiple tasks, such as content generation, report drafting, or data analysis; and is confident in navigating and using the tools without assistance.
Medium	Participant has a functional understanding of LLM AI tools and has used them for basic tasks such as answering queries or drafting simple documents; and is familiar with common functionalities but may not fully explore advanced features.
Low	Participant has limited exposure to LLM AI tools, typically using them only a few times; and may not understand the full potential of the tools and rely on basic functions such as casual querying or one-off tasks.
None	Participant has attempted use of LLM AI tools but refused or discontinue using them after initial attempts.

^a^LLM: large language model.

^b^AI: artificial intelligence.

**Table 2:** Levels of participant’s frequency of LLM^a^ AI^b^ tool use.

Level of frequency of use	Definition
Frequent	Participant uses LLM AI tools as a regular part of their daily or weekly administrative tasks; and integrates the tools into their workflow and consistently relies on them for efficiency.
Occasional	Participants uses LLM AI tools sporadically, typically when prompted by specific needs or tasks; and use is less habitual and more task-driven.
Rare	Participant uses LLM AI tools infrequently; and use is minimal to none.

^a^LLM: large language model.

^b^AI: artificial intelligence.

### Data Analysis

The Colaizzi method was used for thematic analysis. This method was chosen for its structured and systematic approach, which ensures that the findings remain grounded in participants’ experiences while allowing for the extraction of meaningful themes [29]. The steps of analysis are as follows: the research team read the transcripts multiple times to gain an in-depth understanding of the participants’ narratives. Key statements directly related to the research objectives were identified. Significant statements were interpreted to extract underlying meanings, ensuring that they aligned with participants’ perspectives. Related meanings were grouped into broader themes, reflecting patterns and variations in the data. A detailed description of each theme was developed, incorporating illustrative quotes from the data. The themes were synthesized into a cohesive narrative that addressed the research objectives. Finally, the findings were shared with participants to ensure that the results accurately represented their experiences and perspectives. Intercoder coding discrepancies were resolved through internal discussions to reach a consensus. If necessary, coders resorted to the original interview recordings. If the discrepancy persisted, the participants in question were contacted for clarification.

### Study Rigor

We used established trustworthiness measures to ensure the reliability and validity of our findings. Member checking was conducted to validate interpretations with participants. An audit trail documenting all key decisions made during data collection and analysis was maintained. Findings were grounded in participants’ narratives, and researcher biases were minimized through reflexivity. To enhance rigor, we conducted member checking, peer debriefing, and reflexivity discussions during biweekly team meetings, where the research team actively reflected on their preconceptions and biases throughout the study to critically evaluate their influence on the research process and findings.

### Ethical Considerations

The study was exempted from ethical review or approval according to the academic research ethical regulations of the People’s Hospital of Peking University as it did not involve patients or patient data. Participants provided written informed consent after receiving detailed explanations about the study’s purpose, their rights, and the measures to ensure confidentiality. They were also informed that participation was entirely voluntary and assured that they could withdraw from the study at any time without any penalty or impact on their professional roles. Participants’ identities were protected by assigning codes, which were formatted as S) (site number and local participant number, respectively); for example, code S2P3 means participant 3 at site 2. Sensitive details were desensitized in demographic data and transcripts to ensure confidentiality. All demographic data, recordings, transcripts, and field notes were securely stored on password-protected flash drives, which were kept in the local principal investigators’ locked drawers. Access to the data was restricted to the research team members. The data were used solely for analysis and reporting purposes in this study. No compensation was provided to participants.

### Use of LLM AI in This Study

ChatGPT was used for translating the interview guide, participant quotes, and the initial manuscript draft from Chinese to English and polishing the English language in subsequent revisions. All AI-generated contents were reviewed by 2 bilingual researchers to ensure accuracy. No other steps involved the use of LLM AI to ensure originality and maintain the integrity of the research process.

## Results

### Interviews

By the time data saturation was achieved, a total of 31 participants across 3 sites had completed the interviews, including 9 participants at site 1, 10 at site 2, and 12 at site 3, respectively. Interviews lasted an average of 27.3 minutes (range 21-39 minutes).

### Participant Demographics

Most participants (n=21, 74%) were younger than 45 years, with a roughly equal distribution of male (n=15, 48%) and female (n=16, 52%) participants. Nearly half (n=15, 48%) of participants reported between 5 and 10 years of administrative work experience, while 26% (n=8) had more than 10 years of experience. Regarding education, 55% (n=17) of participants held a “master or over” degree, while the remaining 45% (n=14) held “bachelor or below” qualifications.

In terms of familiarity with LLM AI tools, 23% (n=7) reported “high,” 29% (n=9) “medium,” 26% (n=8) as “low,” and 23% (n=7) reported “none.” Regarding use frequency, 26% (n=8) were “frequent” users, 29% (n=9) were “occasional” users, and the remaining 45% (n=14) were classified as “rare” users (Table 3).

Notably, there were site-specific trends in the levels of familiarity and frequency of use of LLM AI tools. At site 1, only 1 participant (S1P5) reported a high level of familiarity with LLM AI tools, and this participant was also the only frequent user at the site. In contrast, site 3 had the highest proportion of participants with high familiarity (S3P1, S3P7, S3P11, and S3P12), all of whom reported frequent use. Site 2 exhibited a more balanced distribution, with participants showing a mix of low, medium, and high familiarity levels and a corresponding range in frequency of use.

Across all sites, frequency of use tended to correlate with levels of familiarity. Participants categorized as having high familiarity consistently reported frequent use, while those with low familiarity or none typically used the tools rarely or occasionally. Individualized participant characteristic data are provided in Multimedia Appendix 2.

While site-specific trends in familiarity and frequency of use were evident, trends in other characteristics, such as age, sex, work experience, and educational level, were less prominent. These patterns warrant further exploration in future quantitative studies to establish causal relationships.

**Table 3:** Demographic characteristics of participants (N=31).

Characteristics						Participants, n (%)
**Site**						
	1				9 (29)	
	2				10 (32)	
	3				12 (39)	
**Age (years)**						
	Less than 35		12 (39)			
	35-45		11 (36)			
	More than 45		8 (26)			
**Sex**						
	Male				15 (48)	
	Female				16 (52)	
**Administrative work experience (years)**						
	Less than 5		8 (26)			
	5-10		15 (48)			
	More than 10		8 (26)			
**Education level**						
	Bachelor or less		14 (45)			
	Master or higher		17 (55)			
**Familiarity with LLM^a^ AI^b^ tools**						
	High			7 (23)		
	Medium			9 (29)		
	Low			8 (26)		
	None			7 (23)		
**Frequency of LLM AI tool use**						
		Frequent				8 (26)
		Occasional				9 (29)
		Rare				14 (45)

^a^LLM: large language model.

^b^AI: artificial intelligence.

### Themes

Three key themes were generated from the interviews, as follows.

#### Theme 1: Factors Influencing Adoption and Continued Use

##### Overview

The adoption and continued use of LLM AI tools were shaped by various individual and contextual factors. Participants’ familiarity with technology, prior knowledge, early experiences with the tools, personality traits, and entrenched work habits often determined their level of engagement and willingness to explore further.

##### Subtheme 1: Initial Knowledge and Tech Literacy

Participants’ understanding of LLM AI tools and their general comfort with technology played a pivotal role in adoption. Those with prior exposure to technology or experience with similar tools tended to adopt them more readily, while those with limited knowledge expressed hesitation or uncertainty.

Participants who lacked awareness or foundational knowledge about LLM AI tools often struggled to take the first step toward adoption. The sample quotes were as follows.

I had no idea these tools existed until a colleague mentioned it during a meeting. I wasn’t sure how it worked, and without any formal introduction or training, it felt intimidating to even try... Technology isn’t my strong suit, and I need some support before I can confidently use something like this.

S1P1

It’s not that I don’t like technology. I just don’t have the prior knowledge to understand it easily... It’s hard to even know where to start.

S2P10

In contrast, participants with prior technological familiarity emphasized how their existing skills made it easier to explore and use LLM AI tools effectively. Participants with a tech-savvy background found it easier to engage with LLM AI tools and highlighted their use in improving workflow efficiency.

I’m generally good with tech, so when I heard about the tools, I right away wanted to explore them. Once I started (using them), I realized how much they could help with daily tasks.

S1P5

People who are already tech-savvy seem to adopt something like these tools much faster. I myself lack understanding the basics of how these tools work。 This was a big challenge. I felt that I was at a disadvantage compared to other younger colleagues. They are more familiar with technologies.

S3P11

##### Subtheme 2: Initial Attempts and Impressions

Participants’ first experiences with LLM AI tools often shaped their ongoing engagement. Early successes motivated continued use, while initial challenges or failures created a reluctance to persist. Participants who had positive first experiences often felt empowered to explore additional applications.

The first time I used an LLM AI tool, I was blown away. I needed to draft a report quickly, and it generated a clear and concise version in seconds, literally second. (I’m) not joking! (Look astonished) The feeling was so positive that I immediately started exploring other ways (to use it)... Now it’s a regular part of my daily routine.

S3P1

Conversely, negative first experiences often discouraged participants, creating a sense of frustration and mistrust in the tools.

My first attempt with the tool didn’t go as expected. The output wasn’t what I expected. I felt very frustrated.

S1P4

I gave it a shot when someone showed me a demo*, but my experience wasn’t as smooth. It seemed too complicated to complete my task. I didn’t feel like I had the time to figure it out. The initial failure discouraged me (from continuing).* [S2P9]

##### Subtheme 3: Personality Traits and Habits

Participants’ attitudes toward change and their existing work habits influenced how they engaged with LLM AI tools. Those who were resistant to change or highly attached to their traditional workflows often avoided experimenting with new tools.

I’m the type of person who likes to stick to what I know. These tools indeed seem interesting, but I don’t feel the need to use them. My current workflow works fine for me. I just don’t see the urgency.

S2P4

Once you’re used to your routine, it’s hard to think about changing it... Just don’t need (the tools) now.

S3P5

In contrast, participants with a natural curiosity for innovation viewed these tools as opportunities for improvement.

Some of us are naturally curious about new technologies, like me. (Laughed) Curiosity drives exploration, you know... I like to experiment with new tools and figure out how they can make my job easier, but it’s true that not everyone is like me.

S1P8

I like trying new things, but I know a lot of my colleagues are hesitant about new stuffs. It’s just a matter of personality or preference.

S2P8

Personally, I enjoy exploring new ways to make my work more efficient, so I’m pretty open to these AI tools.

S3P1

#### Theme 2: Limited Tasks for Which Tools Were Used

Participants predominantly used LLM AI tools for document drafting, while other potential applications remained largely unexplored. This limited range reflected a combination of low awareness, confidence, and lack of training.

##### Subtheme 1: Limited Range of Tasks

While document drafting was the most common use case, participants expressed interest in broader applications if provided with the necessary guidance.

I mostly use them for writing documents, like reports, emails, plans, all kinds of writing basically. I’ve heard they can do much more, but I don’t know how to use other features.

S2P3

##### Subtheme 2: Barriers to Broader Application

This restricted application was further compounded by barriers preventing participants from exploring the full capabilities of the tools, such as a lack of trust in the tools' accuracy for complex tasks, limited skills with crafting effective prompts, and perceptions that the effort required to use the tools outweighed the benefits.

Lack training. I’m stuck with simple tasks. I tried using them for data analysis once, but the output had errors. I couldn’t fix them, so I lost trust in using them for anything beyond basic tasks.

S1P4

It’s easier to stick to what you know the tools can do. When I tried prompting Kimi (a Chinese LLM AI tool) for a more complex task, it didn’t work well. I spent more time rephrasing the prompt and correcting the output than if I had just done it manually. It didn’t feel worthwhile in the end.

S2P7

I tried using it to summarize a report, but it left out key details and added irrelevant information... For important things, like scheduling or analyzing data, I just don’t trust it.

S3P4

#### Theme 3: Desirability of Tutorials and Training

Participants strongly emphasized the need for structured training to improve familiarity, confidence, and the range of applications for LLM AI tools.

A hands-on tutorial would make a huge difference. If someone could walk us through the features step by step, it would be very helpful.

S2P5

(Training) like workshops to show how to use it in real-world scenarios. Right now, we’re just experimenting blindly.

S3P4

Participants also emphasized the importance of institutional responsibility in providing training opportunities.

I think if the hospital can provide some training sessions, more people would be willing to give these tools a chance... Even just a basic introduction to how the tools work and what they can do would be helpful... For many of us, the biggest barrier is not knowing where to start or how to use the tools effectively.

S1P5

Training should include not just how to use the tools but also how to troubleshoot common issues... Definitely help building confidence.

S3P7

Notably, participants of site 3 mentioned that their hospital, as a leading academic center, provided their clinical and administrative staff with lectures about the adoption of LLM AI for research applications.

Not specially for administrative work, our hospital organized lectures about how to use AI for research. I’m not sure if this answers your question but this was helpful for us (administrators) as well.

S3P4

Similar initiatives were not offered at sites 1 or 2, which might partly explain the observed site-specific discrepancies.

## Discussion

### Principal Findings

The adoption of LLM AI tools among hospital administrators was generally low, with notable site-specific variations highlighting the potential influence of regional and institutional contexts. While participants at some sites reported higher familiarity and frequent use, others demonstrated limited engagement, reflecting a mix of interest, barriers, and opportunities. Three key themes emerged from the data: factors influencing adoption and continued use, the limited range of tasks for which the tools were used, and the strong desire for structured tutorials and institutional training. These findings suggest that individual and contextual factors, including prior knowledge, technological literacy, and early user experiences, play a critical role in shaping engagement with LLM AI tools. Furthermore, the restricted application of these tools, coupled with barriers such as mistrust in their accuracy and insufficient prompting skills, underscores the need for targeted interventions to enhance their usability and integration.

According to our findings, the adoption of LLM AI tools among hospital administrators was generally low, with notable variations across sites. Participants at site 3 reported higher familiarity and more frequent use compared to their counterparts at sites 1 and 2. Notably, administrators at site 3 highlighted that their hospital, which is a leading academic center, provided lectures on the use of AI for research applications. This additional initiative may have contributed to their increased familiarity and comfort with AI tools and fostered an encouraging, supportive organizational culture, suggesting that regional and institutional contexts, including proactive educational support, play a significant role in shaping adoption behaviors. The observations align with previous studies indicating that local organizational culture and support systems are critical factors in technology uptake, particularly in health care settings where resources and attitudes toward innovation can vary widely [30-33].

There seemed to be a clear correlation between participants’ levels of familiarity with LLM AI tools and their frequency of use. Across all sites, participants with high familiarity consistently reported frequent use, while those with low familiarity or no prior experience typically engaged with the tools rarely or occasionally. This trend demonstrates the importance of technological familiarity as a key determinant of sustained engagement, suggesting that users who feel confident navigating the tools are more likely to integrate them into their workflows. Similar findings have been observed in studies, where perceived ease of use and prior knowledge significantly influenced the adoption and continued use of technology [34,35].

The adoption and continued use of LLM AI tools were shaped by various individual and contextual factors [36-38]. Participants who were more familiar with technology or had prior experience using similar digital tools the AI tools more readily. Initial knowledge and technological literacy may play a critical role in this process. The readiness to experiment aligns with the Technology Acceptance Model, which asserts that perceived ease of use and prior experience are key determinants in the decision to adopt new technologies [39]. In contrast, participants with limited technological expertise exhibited a greater degree of caution and reluctance and often expressed concerns about the complexity and reliability of the tools. They tended to indicate a need for more accessible resources, such as user-friendly tutorials, comprehensive onboarding sessions, or hands-on training workshops, to demystify the technology and build confidence.

This divergence suggests that individual attitudes and previous exposure may influence initial adoption decisions and affect long-term engagement and satisfaction. For instance, a lack of digital fluency can lead to underuse, as users may find the tools intimidating or prone to errors, thereby reinforcing resistance to change. On the other hand, positive early experiences can reduce apprehension and encourage users to integrate the tools more deeply into their workflows. Consequently, it is important to provide tailored support mechanisms, possibly catering to varying levels of digital proficiency to ensure that the potential benefits of LLM AI tools are accessible to all administrative staff, regardless of their initial technological background. This is also reflected in our findings according to theme 3, where many participants indicated desirability for educational initiatives.

Early experiences with the tools played a pivotal role in shaping overall engagement. Participants who encountered positive outcomes during their initial interactions, such as generating accurate drafts, receiving timely and coherent responses, or completing tasks more efficiently, tended to be reassured of the technology’s potential and motivated for further exploration. Such early successes often served as a catalyst for a deeper integration of the tools into their workflows, which reinforced confidence and sparked curiosity. Conversely, participants who faced challenges during initial attempts, such as difficulties in crafting effective prompts, inconsistent outputs, or errors in task execution, tended to develop skepticism and mistrust toward the technology. The negative experiences not only discouraged further exploration but also reinforced preexisting apprehensions about the reliability and practicality of the tools, particularly for complex or critical tasks. Such experiences often lead to a cycle of avoidance, where initial failures contribute to a diminished willingness to invest time and effort in mastering the technology. This pattern is consistent with prior research, which has shown that early user experiences with technologies significantly impact long-term adoption and sustained use [40,41]. Positive early interactions can lead to a virtuous cycle of increasing confidence and proactive engagement, whereas negative experiences can result in long-lasting reservations and resistance to change.

Individual attitudes and habits emerged as another key factor influencing adoption. Some participants, driven by inherent curiosity and a proactive willingness to experiment, viewed the tools as innovative opportunities to streamline and enhance their workflows. The early adopters were more inclined to explore its diverse functionalities. Their openness often stemmed from personality traits such as adaptability and a high degree of openness to experience, which have been consistently linked with higher rates of technology adoption [42]. In contrast, other participants exhibited a strong preference for established, traditional methods. For them, the idea of adapting to new technological tools was unfamiliar and intimidating. The reluctance was often rooted in a comfort with routine practices and a skepticism toward untested innovations, reflecting lower risk tolerance. Such individuals were less inclined to divert from methods that they perceived as reliable, even when potential efficiency gains were evident.

The interplay between individual preferences and organizational culture further influenced the adoption patterns. In environments where the culture actively encourages innovation, experimentation, and continuous learning, even those initially hesitant to adopt new tools may be motivated to try them out. Conversely, in settings where traditional practices dominate and there is little institutional support for digital transformation, resistance to change can be reinforced. This dynamic is partly evidenced by the site-specific discrepancies in this study, where site 3 offering educational initiatives as a leading academic center may foster a supportive environment for adoption. This is consistent with prior reports stressing the value of a supportive organizational culture may well reduce apprehension and foster experimentation [43,44].

The limited range of tasks for which LLM AI tools were used further illustrates significant barriers to broader adoption. Most participants mainly used the tools for drafting documents, a narrow application suggesting a prevailing lack of awareness, confidence, and adequate training. The conservative use pattern was compounded by a deep-seated mistrust in the tools’ ability to handle complex tasks accurately, as well as by the frustration associated with crafting effective prompts. Several participants noted that the time and cognitive effort required to refine prompts and correct outputs outweighed the potential benefits. As a result, they tended to rely on simpler, more familiar workflows. The challenges hindered the exploration of advanced features and reinforced a cycle of underuse, where negative early experiences and perceived inefficiencies further discouraged experimentation.

Similar observations in existing literature emphasize that the cognitive and time demands of prompt engineering are significant barriers to the efficient use of AI tools [23,45,46]. The findings suggest that without targeted interventions, such as improved user interfaces, streamlined prompt formulation guides, and comprehensive training programs, users may continue to favor basic applications over more sophisticated and potentially transformative functionalities. Again, this pattern stresses the critical need for organizational support and continuous training to build user confidence and optimize overall use.

Participants strongly emphasized the need for structured tutorials and training programs to enhance their understanding and confidence. Suggestions included hands-on demonstrations, real-world application workshops, and resources to improve prompting skills. These findings resonate with prior research advocating for tailored, user-centric training programs to support technology adoption [47,48]. Participants also stressed the importance of institutional support, noting that training sessions integrated into professional development initiatives could normalize tool use and encourage broader engagement.

Based on participants’ feedback and prior reports [47,48], we recommend that institutions develop a comprehensive, multifaceted approach to training and support. Specifically, hospitals should design structured tutorials and hands-on training programs tailored to the needs of administrative staff. For example, institutions could organize web-based workshops that simulate real-world administrative scenarios, provide step-by-step demonstrations on using LLM AI tools, and offer dedicated sessions to improve prompt formulation and troubleshooting skills. In addition, developing accessible digital resources such as video tutorials, user guides, and FAQs can help reinforce learning and serve as a reference for users.

Addressing barriers such as insufficient training, mistrust in accuracy, and limited prompting skills could significantly enhance adoption rates. By prioritizing comprehensive training programs and fostering a culture of innovation, institutions can better support administrators in integrating LLM AI tools into their daily workflows. Current literature suggests that organizational-level strategies, including leadership support and resource allocation, are key to facilitating successful technology adoption [49].

### Limitations

This study has 2 key limitations. First, as a cross-sectional study, it provides a snapshot of adoption behaviors at a single point in time, which limits our ability to explore the over-time evolution of LLM AI tool adoption. Future longitudinal studies may be conducted to better capture changes in familiarity, frequency of use, and the impact of interventions on adoption patterns. Second, as a qualitative study, the findings are descriptive and exploratory in nature. While we observed correlations between participant characteristics, such as familiarity levels and frequency of use, the interpretations are preliminary, and the relationships with other characteristics, such as age, sex, and education, remain undetermined. Future quantitative investigations are needed to confirm the patterns, establish causal relationships, and assess the broader applicability of the findings.

### Conclusions

Familiarity with technology, positive early experiences, and openness to innovation may facilitate adoption, while barriers such as limited knowledge, mistrust in tool accuracy, and insufficient prompting skills can hinder broader use. LLM AI tools are now primarily used for basic tasks such as document drafting, with limited application to more advanced functionalities due to a lack of training and confidence. Structured tutorials and institutional support are needed to enhance usability and integration. Targeted training programs, combined with organizational strategies to build trust and improve accessibility, could enhance adoption rates and broaden tool use. Future quantitative investigations should validate the adoption rate and influencing factors.

## Appendix

Multimedia Appendix 1 Interview guide-with Chinese original.

Multimedia Appendix 2 Individualized participant demographics.

## Abbreviations

AI: artificial intelligence
LLM: large language model

## Data Availability

The audio recordings of the interviews are prohibited from sharing by institutional privacy protection policy. Desensitized interview transcripts may be provided on reasonable request to the corresponding author.
