# Supplementary material for: Adoption of Large Language Model AI Tools in Everyday Tasks: Multisite Cross-Sectional Qualitative Study of Chinese Hospital Administrators
Source: J Med Internet Res. 2025 Apr 1;27:e70789. doi: 10.2196/70789 (PMC12000786; doi:10.2196/70789)
Supplement: Multimedia Appendix 1 [file jmir_v27i1e70789_app1.pdf]

Appendix 1: Interview Guide: Semi-Structured Interview on the Usage of Large Language Model (LLM) AI Tools by Chinese Hospital Administrators

Interview Introduction

Opening Welcome and Research Purpose Introduction:

1. Welcome the participant and self-introduction.
2. Explain the research purpose: "This study aims to explore the experiences of hospital administrators in using LLM AI tools in their daily work. Your feedback will help us understand the factors that influence the use of these AI tools, the usage patterns, and the potential barriers."

Confidentiality and Recording Consent:

1. Assure the participant of confidentiality: "Your responses will be kept strictly confidential, and any identifying information will be anonymized or removed in the data analysis and subsequent publications."
2. Obtain the participant's consent to record the interview.

Part One: Background Information

Role and Responsibilities:

1. Please describe your current position and main responsibilities in the hospital.
2. How long have you worked in this position?

Experience with Technologies:

1. How confident do you feel you are in using various technological tools in your daily work?
2. Please share examples of other digital technology tools you have used in your administrative work.

Part Two: Familiarity with LLM AI Tools

Exposure and Awareness:

1. Have you ever used LLM AI tools, such as ChatGPT or similar tools?
2. If yes: How did you first learn about these tools? Was it through colleagues, organizational events, or did you discover them independently?
3. Based on the following criteria, which best describes your familiarity with LLM AI tools:

   (1) **Very familiar (High):** You have an in-depth understanding of the AI tools, including their advanced features, and are confident in using them for drafting content, writing reports, or data analysis.

(2) **Moderately familiar (Medium):** You understand the basic functions and can use them to answer queries or draft simple documents, but your use of advanced features is limited.

(3) **Somewhat familiar (Low):** You have limited exposure, have only used them a few times for solving basic issues, and lack an in-depth understanding of their potential.

(4) **Not familiar (None):** You have tried these tools but chose not to continue using them, or you have no experience at all.

Understanding of Features:

1. Which specific features of LLM AI tools are you familiar with? Have you tried using these tools to draft reports, analyze data, or assist with scheduling?

Frequency of Use:

1. How frequently do you use LLM AI tools in your administrative work? According to the following criteria, which best describes your usage frequency:

   (1) **Frequent:** Regularly used on a daily or weekly basis for various tasks to enhance efficiency.

   (2) **Occasional:** Used sporadically, typically for specific needs or tasks.

   (3) **Rare:** Used very infrequently or only occasionally, often due to certain obstacles or organizational constraints.

2. Has your usage frequency changed over time? What factors have influenced this change?

Part Three: Adoption and Application

Early Experiences:

1. Can you recall your first experience using an LLM AI tool?
2. Was that experience positive or challenging (negative)? How did it affect your subsequent use of these tools?

Integration into Work Processes:

1. Have you integrated AI tools into your daily work processes?
2. In which specific tasks do you find these AI tools most useful? Could you provide examples?

Perceived Benefits:

1. What benefits have you experienced from using AI tools? Have they improved your work efficiency or workflow?

Part Four: Barriers and Challenges

Factors Hindering Adoption:

1. What difficulties have you encountered when trying to use AI tools?
2. Are there any specific tasks where the AI tools have not met your expectations?

Prompting Skills:

1. How confident are you in formulating prompts to obtain accurate and useful responses from these tools?
2. Have you encountered any difficulties in designing prompts or improving response accuracy, especially for complex work tasks?

Institutional Factors:

1. Does your organization encourage the use of AI tools? Are there any organizational factors that hinder the adoption of AI tools?

Part Five: Suggestions and Future Needs

Training and Support:

1. What types of training or support would help you use AI tools more effectively?
2. Would structured tutorials, workshops, or guidance on specific functions be beneficial to you?

Tool Improvements:

1. Which features of AI tools do you think could be further optimized to better assist your work?
2. Regarding the future development of AI tools, what unmet needs do you think should be addressed?

Interview Conclusion

Summary of Key Points:

1. Briefly summarize the participant's core viewpoints and ask if they have anything further to add.

Thank You and Follow-Up:

2. Thank the participant for their participation and valuable feedback.
3. Provide contact information for any follow-up questions or further feedback.

Chinese original of interview guide:

访谈指南：关于中国医院行政管理人员对大语言模型人工智能工具使用情况的半结构化访谈

访谈前言

开场欢迎，研究目的介绍：

1. 欢迎受访者，访谈者自我介绍。
2. 解释研究目的："本研究旨在探讨医院行政管理人员在日常工作中使用大语言模型 AI 工具的感受，您的反馈将帮助我们了解影响管理人员使用这些 AI 工具中的影响因素、使用模式，以及潜在障碍。"

保密性与录音同意：

1. 向受访者保证研究保密性："我们对您的回答内容将严格保密，在数据分析和后期发表文章中可能涉及的所有身份信息都会做脱敏处理或者直接删除。"
2. 获得受访者的同意对访谈进行录音。

第一部分：背景信息

角色与职责：

1. 请描述一下您在医院目前的职位和主要职责。
2. 您在这个职位上工作多久了？

技术工具使用经验：

1. 您感觉您在日常工作中使用各种技术工具的熟练程度如何？
2. 请您讲讲在行政工作中，您使用过的其他数字技术工具的例子。

第二部分：对大语言模型 AI 工具的熟悉程度

接触与认知：

1. 您是否曾使用过 LLM AI 工具，例如 ChatGPT 或类似工具？
2. 如果回答"是"：您最开始是如何了解到这些工具的？是同事介绍、单位活动，还是您自己发现的？
3. 按照下面的标准，请问哪一项最能描述您对大语言模型 AI 工具的熟悉程度：

   （1）非常熟悉：对 AI 工具有深入了解，包括其高级功能，并有信心将其用于草拟内容、报告撰写，或数据分析等多种任务。

   （2）中等熟悉：了解基本功能，可用来答疑解惑或草拟简单文件，但对高级功能的使用有限。

（3）一般熟悉：接触有限，仅使用过几次，解决一些浅显基础的问题，对其潜力缺乏深入了解。

（4）不了解：曾尝试使用过这些工具，但选择不继续使用，或者完全没有使用经验。

对功能的了解：

1. 您熟悉 LLM AI 工具的哪些具体功能？您是否尝试过使用其功能来起草报告、分析数据或协助日程安排？

使用频率：

1. 您在行政管理工作中使用大语言模型 AI 工具的频率如何？按照下面的标准，请问哪一项最能描述您的使用频率：

（1）频繁使用：日天或每周经常用于各种任务，使用其提高效率。

（2）偶尔使用：使用较为零散，通常是用于特定需求或任务。

（3）很少使用：使用的很少或这仅仅偶尔使用，通常是由于存在某些阻碍或者单位条件不允许。

2. 您的使用频率有没有随着时间发生变化？哪些因素影响了这种变化？

第三部分：接受与应用

早期体验：

1. 您能回忆一下第一次使用 LLM AI 工具的经历吗？
2. 这次经历是积极的，还是让您感到有挑战性的（负面的）？对您后续继续使用这些工具有哪些影响？

与业务流程结合：

1. 您是否将 AI 工具结合到您的日常工作流程当中了？
2. 您觉得在哪些具体工作任务中，这些 AI 工具最有用？能否举例说明？

感受到的好处：

1. 使用 AI 工具给您带来了哪些好处？是否改善了您的工作效率或流程？

第四部分：阻碍与挑战

阻碍应用的因素：

1. 在尝试使用 AI 工具的过程中，您遇过哪些困难？
2. 有没有哪些具体的任务，AI 工具没能达到您的预期？

提示技能：

1. 在设计提示语，以获取准确且有用的回答这方面，您感觉有信心吗？
2. 您在制定提示语或者提高回答准确性方面有没有遇到什么困难，特别是在用于复杂的工作任务时？

机构因素：

1. 您单位是否鼓励使用 AI 工具呢？有没有什么跟单位有关的因素，对使用 AI 工具存在阻碍？

第五部分：建议与未来需求

培训与支持：

1. 哪些类型的培训或者支持可以帮助您更有效地使用 AI 工具？
2. 组织指导课、研讨会，或者针对特定功能的指导，是否会对您有所帮助？

工具改进：

1. 您觉得 AI 工具中的哪些功能还可以进一步优化，可以进一步帮助您的工作？
2. 针对未来 AI 工具的开发中，您觉得还有哪些没有照顾到的需求？

访谈结尾

总结要点：

1. 简要总结受访者的核心观点，并询问对方是否有要补充的内容。

感谢与后续：

2. 感谢受访者的参与和宝贵意见建议。
3. 提供联系方式，以便受访者有后续问题或反馈时联系。
