# Supplementary material for: Adoption of Large Language Model AI Tools in Everyday Tasks: Multisite Cross-Sectional Qualitative Study of Chinese Hospital Administrators
Source: J Med Internet Res. 2025 Apr 1;27:e70789. doi: 10.2196/70789 (PMC12000786; doi:10.2196/70789)
Supplement: Multimedia Appendix 2 [file jmir_v27i1e70789_app2.docx]

**Appendix 2: Individualized Participant Demographics**

| **Participant Code** | **Age** | **Gender** | **Years of Administrative Work Experience** | **Education Level** | **Familiarity with LLM AI Tools** | **Frequency of LLM AI Tool Use** |
| --- | --- | --- | --- | --- | --- | --- |
| S1P1 | <35 | Male | 5-10 | Bachelor or below | Low | Rare |
| S1P2 | 35-45 | Female | >10 | Master or over | Low | Rare |
| S1P3 | <35 | Female | <5 | Bachelor or below | Medium | Occasional |
| S1P4 | >45 | Male | >10 | Master or over | Low | Occasional |
| S1P5 | <35 | Female | <5 | Bachelor or below | High | Frequent |
| S1P6 | 35-45 | Female | 5-10 | Master or over | None | Rare |
| S1P7 | <35 | Male | <5 | Bachelor or below | None | Rare |
| S1P8 | >45 | Female | >10 | Master or over | Medium | Frequent |
| S1P9 | 35-45 | Male | <5 | Master or over | Medium | Occasional |
| S2P1 | 35-45 | Female | 5-10 | Bachelor or below | Low | Rare |
| S2P2 | <35 | Female | <5 | Master or over | None | Rare |
| S2P3 | 35-45 | Female | 5-10 | Bachelor or below | Low | Occasional |
| S2P4 | >45 | Male | >10 | Master or over | Low | Rare |
| S2P5 | 35-45 | Female | 5-10 | Bachelor or below | Medium | Frequent |
| S2P6 | <35 | Male | <5 | Master or over | None | Rare |
| S2P7 | >45 | Female | >10 | Master or over | Medium | Occasional |
| S2P8 | 35-45 | Male | 5-10 | Bachelor or below | Medium | Frequent |
| S2P9 | <35 | Female | <5 | Master or over | Low | Rare |
| S2P10 | <35 | Female | <5 | Bachelor or below | None | Rare |
| S3P1 | 35-45 | Female | 5-10 | Master or over | High | Frequent |
| S3P2 | <35 | Male | <5 | Bachelor or below | Medium | Rare |
| S3P3 | >45 | Female | >10 | Master or over | Low | Rare |
| S3P4 | <35 | Male | 5-10 | Bachelor or below | Medium | Occasional |
| S3P5 | >45 | Male | >10 | Master or over | None | Rare |
| S3P6 | 35-45 | Male | 5-10 | Bachelor or below | Low | Rare |
| S3P7 | <35 | Male | <5 | Master or over | High | Frequent |
| S3P8 | >45 | Female | >10 | Bachelor or below | Low | Occasional |
| S3P9 | 35-45 | Female | <5 | Master or over | Low | Occasional |
| S3P10 | 35-45 | Female | 5-10 | Master or over | Low | Rare |
| S3P11 | 35-45 | Female | >10 | Bachelor or below | High | Frequent |
| S3P12 | <35 | Male | <5 | Master or over | High | Frequent |
